# Pharmacotoxicology of Non-fentanyl Derived New Synthetic Opioids

**DOI:** 10.3389/fphar.2018.00654

**Published:** 2018-06-20

**Authors:** Renata Solimini, Simona Pichini, Roberta Pacifici, Francesco P. Busardò, Raffaele Giorgetti

**Affiliations:** ^1^National Centre on Addiction and Doping, Istituto Superiore di Sanità, Rome, Italy; ^2^Unit of Forensic Toxicology, Department of Anatomical, Histological, Forensic and Orthopedic Sciences, Sapienza University of Rome, Rome, Italy; ^3^Section of Legal Medicine, Università Politecnica delle Marche, Ancona, Italy

**Keywords:** novel synthetic opioids, U-47700, U-49900, AH-7921, MT-45, toxicity, health threat

## Abstract

A class of opioid agonists not structurally related to fentanyl, derived from research publications of pharmaceutical companies or patents within the United States and abroad are contributing to the current opioid epidemic. Novel synthetic opioids (NSOs) created to circumvent drug control laws such as U-47700, U-49900, AH-7921, or MT-45 have no recognized therapeutic use, are clandestinely manufactured and sold on conventional or dark web. We herein provide a review of the pharmacological properties available on most of these substances trying to provide a better knowledge on these compounds, particularly with respect to toxicity and dangerous adverse effects in users. Indeed, these NSOs share not only a great potency of action and receptor affinity with respect to natural or synthetic opiates (e.g., morphine, heroin, and methadone) but also a non-negligible toxicity leading to intoxications and fatalities, posing a serious harm to public health and society.

## Introduction

Non-fentanyl derived novel synthetic opioids (NSOs) have initially emerged worldwide as non-illegal drugs diffused to replace heroin and thus circumvent prohibition laws, resulting in numerous abuse reports and overdose cases, especially across United States and Europe (Carroll et al., 2012; Armenian et al., 2017b; Baumann et al., 2017; Fabregat-Safont et al., 2017).

These NSOs are a broad family of analgesics and anesthetics, mainly synthesized in the 1970s, acting at the mu (μ) opioid receptor, but also at the delta (δ) and kappa (κ) ones. The power of physiological and psychological effects is different according to the specific synthetic opioid being used and the type of receptor that is activated or inhibited (Baumann et al., 2017; European Monitoring Centre for Drugs, and Drug Addiction [EMCDDA], 2017).

To precisely define this particular class of NSOs, it is worth mentioning that the alkaloid compounds naturally found in the opium poppy plant are defined opiates and include, among others, morphine, codeine, and thebaine as principal alkaloids.

Instead, substances such as hydromorphone, oxymorphone, and heroin are semisynthetic opioids made from morphine with pharmacological properties similar to those of opiates and affinity for one of the 7-transmembrane G protein-coupled opioid receptors (Raffa et al., 2018).

All the above reported opioids belong to the phenanthrene family, while the family of benzomorphans include, e.g., pentazocine phenazocine, dezocine, and eptazocine, developed through the modification of the basic phenanthrene structure of morphine (Cittern et al., 1986). Conversely, methadone is a phenylheptylamine agent whereas meperidine is a phenylpiperidine derivative (Knapp, 2002; Raffa et al., 2018).

The family of phenylpiperidines (characterized by a phenyl moiety directly linked to a piperidine) includes also the NSO fentanyl (synthesized by P. Janssen in the 1960s) and its analogs, up to 1000 times more potent as analgesic than meperidine and differing in structure from the latter for a phenethyl group on the piperidine nitrogen in place of a methyl group (Elbaridi et al., 2017; Raffa et al., 2018).

While extensive literature has been published in regards to pharmacology and toxicology of fentanyl and its illicit analogs (Bäckberg et al., 2015; Mounteney et al., 2015; Dwyer et al., 2017; Giorgetti et al., 2017; Guerrieri et al., 2017; Helander et al., 2017a; Pichini et al., 2017a,b; Shoff et al., 2017; Suzuki and El-Haddad, 2017), the pharmacological and toxicological properties of non-fentanyl derived NSOs have not yet been reviewed in detail.

Compounds such as U-47700, U-51754, U-49900, U-448800, AH-7921 from the chemical family of benzamide, U-50488 and U-51754 from the acetamide family and MT-45 from the piperazine family are the NSOs most recently reported as health threats for opioids consumers (Mohr et al., 2016; Amin et al., 2017; Baumann et al., 2017; Domanski et al., 2017; Fabregat-Safont et al., 2017; Prekupec et al., 2017; Marchei et al., 2018). Indeed, this new generation of derivatives has been involved in a number of recent overdose deaths worldwide (Drug Enforcement Administration [DEA], 2016; Baumann et al., 2017; Domanski et al., 2017; Fabregat-Safont et al., 2017).

Clandestine manufacturing of NSOs has been pirated from scientific literature or patent filings published by pharmaceutical companies attempting to search for new therapeutic drugs without addiction-related adverse effects (Logan et al., 2017).

In a similar manner to fentanyl derivatives, these NSOs are being partly used as heroin adulterants or as constituents of counterfeit pain pills and they can be bought directly by users from online vendors via conventional web or cryptomarket (European Monitoring Centre for Drugs, and Drug Addiction [EMCDDA], 2016; Armenian et al., 2017b; Baumann et al., 2017; Van Hout and Hearne, 2017).

Similarly to morphine and heroin (opiates) or to semi-synthetic opioids (like hydro- and oxycodone, hydro- and oxymorphone), these compounds produce CNS depressants effects such as respiratory depression, analgesia, hypothermia, sedation, euphoria, anxiety, sweating, disorientation, drowsiness, nausea, and miosis (Carroll et al., 2012; Guerrini et al., 2013; Hill and Thomas, 2016; Armenian et al., 2017b), and although the effects of tolerance and dependence may rapidly reach high levels, elevated risks of overdose and death are frequent for these compounds (United Nations Office on Drugs and Crime [UNODC], 2017b). Furthermore, the typical rewarding characteristics and the easy availability induce users to abuse of these opioids (Carroll et al., 2012).

The main NSOs AH-7921, MT-45, and U-47700 have been identified in Europe between 2013 and 2016, and over 40 deaths were reported to the European Monitoring Centre for Drugs and Drug Addiction in a short time after that AH-7921 and MT-45 were found out on the European drug market (EMCDDA) (European Monitoring Centre for Drugs, and Drug Addiction [EMCDDA], 2017). Moreover, in 2016 U-47700 has been the cause of at least 46 confirmed fatalities as well as the subject of 88 reports from forensic laboratories submissions in the United States (Fabregat-Safont et al., 2017).

Since the popularity of these substances is rapidly increasing and evolving over time, there is a great need to update all possible information, particularly with respect to their subjective and side effects and to tackle unsolved issues, including limited analytical methods to disclose and monitor different compounds (Katselou et al., 2015; Lucyk and Nelson, 2017).

**FIGURE 1:**
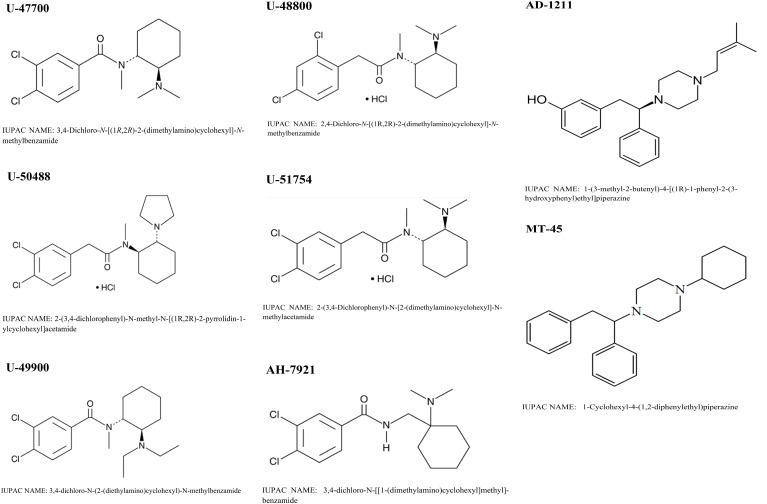
Chemical structures of NSO_s_ reported in this review.

To fill this gap, we here sought to report the latest information available on non-fentanyl derived NSOs U-47700, U-50488, U-51754, U-49900, U-48800, AH-7921, and MT-45 with particular regard to their pharmacotoxicology and adverse effects on users (see **Figure 1**).

### Literature Search

A literature search was performed on the multidisciplinary research databases Scopus and Web of Science and on PubMed for biomedical literature, to identify all the relevant articles (up to March 2018). The search terms used in different combinations were: *new* or *novel synthetic opioid*, *designer opioid/drug,* *analgesics*, *narcotics,* *street drug, novel or new psychoactive substance/drug*. Articles related to fentanyl and its derivatives were excluded. Further studies were retrieved by hand search through the reference lists of the selected articles. Moreover, a search for reports was conducted on Institutional websites, to identify documentation published by international agencies or institutions such as World Health Organization (WHO), United Nations Office on Drugs and Crime (UNODC), United States Drug Enforcement Administration (DEA), and European Monitoring Centre for Drugs and Drug Addiction (EMCDDA). Only articles or reports written in English were selected. All articles were screened independently by three of the authors to determine their relevance in the framework of the current review and only those selected at least by two of them were included.

### NSOs of Benzamide Family

#### U-47700 and U-48800

U-47700 (3,4-dichloro-*N*-[(1R,2R)-2-(dimethylamino) cyclohexyl]-*N*-methylbenzamide), also known under the street names of Pinky (because impurities in its synthesis cause the drug powder to be slightly pink in color), U4 or Fake morphine, is an example of a non-fentanyl benzamide compound initially individuated as a heroin adulterant and as constituent of counterfeit analgesic pills, mimicking pharmaceutical opioids (Drug Enforcement Administration [DEA], 2016; World Health Organization [WHO], 2016; Baumann et al., 2017; Prekupec et al., 2017).

U-47700 is also actively being used as a legal substitute of illegally abused morphine, heroin, or fentanyl derivatives (Coopman et al., 2016).

It is a potent μ-opioid receptor agonist belonging to the *trans-*1,2-diamine class of analgesics and derived from another opioid analgesic compound, AH-7921 (Coopman et al., 2016; Domanski et al., 2017).

U-47700 was originally developed by the Upjohn Company in 1978 and is about 1/10 as potent as fentanyl and 7.5-fold more potent than morphine in animal models.

Up to now, the compound has never been studied in humans and it is not registered for medical use, but possibly induces typical opioid side effects, including respiratory depression, pinpoint pupils, cyanosis, depressed consciousness, and sedation (World Health Organization [WHO], 2016, 2017; Domanski et al., 2017; Prekupec et al., 2017).

It is likely to be used for its morphine-like pharmacological effects such as varying degrees of sedation, euphoria, a general lift in mood with desired effects being experienced in waves. Consumers also report having experimented a “cool, relaxed” effect (Elliott et al., 2016; Domanski et al., 2017).

The routes of administration, as referred by users in web forums, include the oral, insufflation, intravenous and rectal routes and via an inhaler which contains a liquid solution with a minty taste (World Health Organization [WHO], 2016, 2017). Naive information, always on websites, reports that light doses range from 5 to 7.5 mg, common doses from 7.5 to 15 mg and strong doses from 15 to 25 mg. Onset of action after oral administration is around 15 min, duration of subjective effects is 5–7 h and after effects 1–4 h. Similarly, in case of insufflation, onset of action is 15 min, duration of subjective effects is 3–4 h and hang-over period 1–4 h. Finally in case of intravenous use, onset of action is 0–1 min, duration of subjective effects 1–2 h and after effects 1–4 h (Zawilska, 2017).

*In vitro* metabolic profile of U-47700 was recently mapped for the first time using human liver microsomes (HLMs). Found metabolites were *in vivo* verified by analysis of urine specimens collected after five analytically confirmed cases of overdose from U-47700 consumption.

A total of four metabolites were identified in urine specimens. *N*-Desmethyl-U-47700 was recognized as the principal metabolite of U-47700, while the other detected metabolites were *N,N*-didesmethyl-U-47700, *N*-Desmethyl-hydroxyl-U-47700, and *N,N*-Didesmethyl-hydroxyl-U-47700. The study identified also similarities in metabolic transformation between U-47700 and its analog U-49900, resulting in a common metabolite 3,4-dichloro-*N*-(2-aminocyclohexyl)-*N*-methyl-benzamide and isomeric species (Krotulski et al., 2017).

The subjective effects of U-47700 makes it particularly appealing to users when compared to other substances. Indeed, consumers described it as producing more euphoric effects than other fentanyl analogs, more potent in its action than AH-7921, cheap, and easily available (Fabregat-Safont et al., 2017). Users also report the induction of tolerance and the emergence of withdrawal signs and symptoms upon discontinuing use of this compound, being this occurrence suggestive of physical dependence (World Health Organization [WHO], 2016, 2017).

However, abuse of the drug often happens unknowingly to the user, unaware of what he/she is consuming or in other cases the substance is encountered in combination with other drugs (heroin, fentanyl, and fentanyl analogs). Since substances like U-47700 are often produced in illegal laboratories, the identity, purity, and effective dose of the product are unknown (Drug Enforcement Administration [DEA], 2016).

In Belgium a seizure of ‘spice-like’ incense found out, after toxicological analysis, the presence of U-47700 in the herbal mixture. This finding generated great concern, since users appeared to be not aware that they were consuming such a substance openly sold on the Internet as “legal high” (Coopman and Cordonnier, 2017).

Users abusing U-47700 appear to overlap with the individuals abusing prescription opioid analgesics, ‘designer opioids’ or heroin, as evidenced by drug use history documented in U-47700 fatal overdose cases (Drug Enforcement Administration [DEA], Department of Justice, 2016).

A number of fatalities and non-fatal intoxications from U-47700 have occurred in United States and Europe (Coopman et al., 2016; Elliott et al., 2016; Mohr et al., 2016; Armenian et al., 2017b; Domanski et al., 2017; Ellefsen et al., 2017; Jones et al., 2017; McIntyre et al., 2017; Rao and Nelson, 2017; Rambaran et al., 2017; Schneir et al., 2017; Seither and Reidy, 2017; Shoff et al., 2017), with concentrations varying widely, ranging from 7.6 to 1,460 ng/mL; while in other 16 confirmed fatalities across United States, blood concentrations ranged from 17 to 490 ng/mL (Mohr et al., 2016; Logan et al., 2017; World Health Organization [WHO], 2017). In 2016, U-47700 was identified for the first time in East and South-East Asia (United Nations Office on Drugs and Crime [UNODC], 2017a).

Reported symptoms of non-fatal intoxication included respiratory depression, agonal breathing, cyanosis, pinpoint pupils, bilateral pulmonary consolidation, atelectasis, anxiety, nausea, abdominal pain, shivering (Elliott et al., 2016; Armenian et al., 2017a; Domanski et al., 2017; Fleming et al., 2017).

Fatal intoxications have been mostly attributed to cardiac arrest, pulmonary and cerebral edema, cardiomegaly and to the depressant effect on the central nervous system, notably causing respiratory depression (Elliott et al., 2016; Fleming et al., 2017).

An additional compound analog of U-47700 recently emerged on the web is U-48800 (3,4-Dichloro-*N*-[(1R,2R)-2-(dimethylamino)cyclohexyl]-*N*-methylbenzamide). Available information is from drug fora (Bluelight, Reddit) or from research chemicals vendors on the web. Indeed, this agent is available as a research chemical of the opioid analgesic class to replace U-47700. U-48800 was also developed by the Upjohn company in the 1970s and it acts as a selective agonist of the μ-opioid receptor and has around 7.5-fold the potency of morphine in animal models.

U-48800 became the lead compound of selective kappa-opioid receptor ligands such as U-50488, U-51754 (containing a single methylene spacer difference) and U-69593, which share very similar structures. Although not used medically, the selective kappa ligands are used in research (Research chemical, 2018).

#### U-49900

U-49900 (3,4-dichloro-*N*-(2-(diethylamino)cyclohexyl)-*N*-methylbenzamide) is a structural analog of U-47700 with sparse clinical data available. The structural similarities with U-47700 raises concern regarding the risks associated with U-49900 use. Currently, no reported deaths have been associated with U-49900, but this agent is growing in popularity as a replacement or an alternative to the scheduled U-47700. Worldwide use is increasing as it has been specifically documented in some European countries such as Sweden and Spain (Alzghari et al., 2017).

Although both substance names are very similar, U-49900 does not pertain to the same Upjohn patent as the one already mentioned for U-47700 or U-51754, and it is in fact a completely new synthetic opioid (Fabregat-Safont et al., 2017).

Similarly to what happened with U-47700, Krotulski et al. (2017) mapped for the first time the generation of *in vitro* metabolic profile of U-49900 using HLMs. Metabolites were confirmed *in vivo* by analysis of human urine specimens collected after one case report of overdose following U-49900 ingestion. In urine specimens, five metabolites of U-49900 were overall identified. *N*-Desethyl-U-49900 was established to be the main metabolite of U-49900 following microsomal incubations, while *N,N*-didesethyl-*N*-desmethyl-U-49900 was the most abundant in another urine specimen: the other identified metabolites were *N,N*-Didesethyl-U-49900, *N*-Desethyl-hydroxyl-U-49900 and *N*-Desethyl-*N*-desmethyl-U-49900.

As previously mentioned, U-47700 and U-49900 metabolize to a common metabolite 3,4-dichloro-*N*-(2-aminocyclohexyl)-*N*-methyl-benzamide, and this is an important information for the analysts, especially in case of increasing prevalence of U-49900 in the street drug scenario (Krotulski et al., 2017).

U-49900 firstly appeared online in 2016 on a popular drug forum^1^ by a drug user reporting its availability and asking other users about possible dangerous effects. Since the substance is a close analog to U-47700, users expressed concerns about its potential health risks, such as harms caused by nasal and rectal passages, damaged veins, severe withdrawals, and other serious side effects such as loss of taste, smell and of the sense of touch, pain upon insufflation, neurologic pain on the left side of the body and a foam-like discharge from the lungs. In a Swedish forum, a consumer reported usual opioid effects when using a U-49900 dose of 50 mg of the drug intravenously, while at lower doses of 5–10 mg other users informed on no effects. Conversely, in those latter amounts (5–10 mg) U-47700 is already active. Hence, U-49900 needs to be probably consumed at higher doses to have significant effects (Fabregat-Safont et al., 2017).

#### AH-7921

AH-7921 (3,4-dichloro-*N*-{[1-(dimethylamino)-cyclohexyl] methyl}benzamide) is an opioid structurally similar to U-47700 and firstly developed by Allen and Hanburys in the mid-1970s, with extensive *in vitro* and in animal studies, but it has never made available for medical use, because of its heavy addictive properties. AH-7921 was firstly identified in 2012 in samples of a product known as Doxylam which was used by Internet retailers as an alternative name for AH-7921. The name Doxylam could be easily confounded with the name of an antihistamine drug with sedative-hypnotic properties, doxylamine, present in several over-the-counter medicines. The accidental use of AH-7921/doxylam for the treatment of allergy or as a hypnotic might lead to serious health damages (Zawilska, 2017). There is therefore a concern that individuals looking for obtaining the unrelated hypnotic ‘Doxylamine’ might accidentally purchase AH-7921, mislabeled as ‘Doxylam,’ which could lead to unintentional drug overdoses (European Monitoring Centre for Drugs, and Drug Addiction [EMCDDA], 2014).

AH-7921 recently entered the illicit drug market as new psychotropic substance in countries such as Japan, United States and Europe, resulting in several fatalities and intoxications (Karinen et al., 2014; Kronstrand et al., 2014; Coppola and Mondola, 2015; Coopman et al., 2016; Armenian et al., 2017b; Dolengevich-Segal et al., 2017; Domanski et al., 2017; Fels et al., 2017). In fatalities occurred in European countries, the reported blood concentrations ranged from 31 to 1,449 ng/mL (Logan et al., 2017).

AH-7921 is an agonist of μ and κ opioid receptors, with a moderate selectivity toward μ opioid receptors, a narrow therapeutic window, and may cause dependence (Zawilska and Andrzejczak, 2015). It is 1.7-fold more potent than morphine at inducing respiratory depression in mice, suggesting greater risk for adverse effects in humans (Prekupec et al., 2017; Tracy et al., 2017).

AH-7921 can be purchased on the web market under the guise of being a research chemical ‘not for human consumption’ and it has also been detected in synthetic cannabinoid products (Karinen et al., 2014; Fabregat-Safont et al., 2017). Wohlfarth et al. (2016) comprehensively studied AH-7921 metabolism, by assessing HLM metabolic stability and determining AH-7921 metabolic profile after human hepatocytes incubation. Then, the findings in a urine case specimen were confirmed and results were compared to *in silico* predictions. Twelve AH-7921 metabolites after hepatocyte incubation were identified, mainly generated by demethylation, less dominantly by hydroxylation, and combinations of different biotransformations. The two major metabolites after hepatocyte incubation, also identified in the urine case specimen, were desmethyl and di-desmethyl AH-7921. Together with the glucuronidated metabolites, these are likely suitable analytical targets for documenting AH-7921 intake (Wohlfarth et al., 2016).

Users describe its effects to be similar to the classical opioids’ ones including euphoria, mental relaxation, pleasant mood lift; while the side effects include sedation, miosis, nausea, vertigo, hypertension, tachycardia, respiratory depression, hypothermia, and withdrawal symptoms possibly worse than morphine due to its much longer half-life (Coppola and Mondola, 2015; Katselou et al., 2015; Fabregat-Safont et al., 2017).

The substance is sold in the form of capsules, tablets or powder and administration routes described in the web forums include mainly the oral, but also inhaled (vaporized), intravenous, intranasal, sublingual and intrarectal routes with a high risk of overdose (Coppola and Mondola, 2015; Katselou et al., 2015; Dolengevich-Segal et al., 2017). Light doses are from 5 to 10 mg, common doses from 10 to 25 and strong doses > 25 mg. Onset of action for oral administration is 15–45 min, duration 6–8 h and after effects 1–6 h (Zawilska, 2017).

In 2013, AH-7921 was detected in several cases of acute non-fatal intoxications and deaths in United States and European countries such as in Sweden, United Kingdom and Norway, combined with other substances such as cannabis, alcohol, synthetic cathinones, benzodiazepines, metoxetamine, or gabapentin. Lung edema was evidenced during the autopsy in most of the dead people (Coppola and Mondola, 2015; Katselou et al., 2015; Dolengevich-Segal et al., 2017).

In all the reported fatalities, cause of death could be attributed to respiratory depression. In a specific case, the autopsy revealed cerebral edema with moderate to increased intracranial pressure. Moreover, signs for an incipient pneumonia in the central lung sections were found (Fels et al., 2017). The absence of pharmacokinetic and pharmacodynamic information in humans makes the risk related to AH-7921 consumption combined with other central nervous system depressants unknown. Currently available information confirms that AH-7921 is a potent respiratory depressant with a high addictive potential (Coppola and Mondola, 2015).

### NSOs of Acetamide Family

#### U-50488, U-51754

Information about U-50488 and U-51754 is quite scant. These acetamides are U-47700 related compounds, being part of the *trans*-1,2-diamine opioid analgesic chemical class synthesized by the Upjohn Company in the attempt to produce a non-addicting analgesic as potent as morphine (Mohr et al., 2016; Amin et al., 2017; Domanski et al., 2017; Fleming et al., 2017).

U-50488 (2-(3,4-dichlorophenyl)-*N*-methyl-*N*-[(1R,2R)-2-pyrrolidin-1-ylcyclohexyl]acetamide) is a κ-opioid receptor agonist (KOR) with analgesic properties and some reported μ-opioid receptor respiratory antagonist effects (Mohr et al., 2016; Baumann et al., 2017; Domanski et al., 2017).

In animal models, U-50488 has been studied for its diuretic, antitussive, analgesic and anticonvulsant properties, but it is known to induce dysphoria and stress-like effects in rodents (Muschamp et al., 2012; Mohr et al., 2016; Amin et al., 2017). U-50488 abuse potential is unknown and at present this synthetic opioid is an uncontrolled substance available online from companies selling research chemicals (Mohr et al., 2016). Currently, information about the toxicological profile and toxicoepidemiology of U-50488 is poor, although the structural similarity of U-50488 to U-47700 poses users at potential health risks associated with its abuse and easy accessibility (Amin et al., 2017).

U-51754 (*trans-*3,4-dichloro-*N*-[2-(dimethylamino)cyclo hexyl]-*N*-methyl-benzeneacetamide) derived from the same Upjohn patent, has also recently appeared on the market. This substance is not as selective for KOR, and with respect to the effects, consumers report that it is more dysphoric and dissociating than U-47700 (Fabregat-Safont et al., 2017).

### NSOs of Piperazine Family

#### MT-45

MT-45 (1-cyclohexyl-4-(1,2-diphenylethyl)piperazine) is a piperazine derivative chemically unrelated to other opioid agonists, originally synthesized in the 1970s in a Japanese laboratory as an analgesic agent. It is an agonist of κ, μ and δ opioid receptors, with analgesic and sedative effects, with a potency nearly identical to morphine and highly addictive potential, although it has not been studied in human (Lindeman et al., 2014; Papsun et al., 2016; Baumann et al., 2017; Dolengevich-Segal et al., 2017; Logan et al., 2017). In animal studies MT-45 showed a high toxicity (Montesano et al., 2017).

MT-45 has been associated with a number of deaths in United States and Europe (especially in Sweden) (Bradley et al., 2016; Papsun et al., 2016; Baumann et al., 2017; Fels et al., 2017; Logan et al., 2017; Montesano et al., 2017). In 2016, MT-45 was identified for the first time in East and South-East Asia (United Nations Office on Drugs and Crime [UNODC], 2017a).

The light doses orally used range from 30 to 45 mg, the common from 45 to 60 mg and the strong > 60 mg. Onset of action is 30–45 min, duration is 4–6 h and after effects 2–3 h (Zawilska, 2017).

MT-45 blood concentrations in the reported deaths ranged from 8.3 to 1,989 ng/mL; while in a few non-fatal intoxications MT-45 was detected in blood at 6–157 ng/mL (Logan et al., 2017).

A recent study by Montesano et al. (2017), identified the chemical structures of 14 Phase I and II MT-45 metabolites, using primarily the prediction *in silico*, then the metabolites were confirmed by *in vivo* experiments. The detected metabolites are principally products of mono- or dihydroxylation, and *N*-dealkylation; in addition it was observed also a glucuronide conjugation of mono- and dihydroxylated metabolites. Hydroxylated MT-45 showed to be bioactive and may contribute to the overall pharmacotoxicological profile of MT-45 *in vivo*. The knowledge of Phases I and II MT-45 metabolite structure is necessary to develop analytical methods to detect MT-45 for clinical and forensic purposes (Montesano et al., 2017).

MT-45 surfaced on internet shops late 2012 (Fabregat-Safont et al., 2017) and was first reported as a new psychoactive substance (NPS) through the Early Warning System of the EMCDDA in December 2013 (Armenian et al., 2017b). Internet suppliers and retailers typically sell MT-45 in its dihydrochloride salt form. It has been seized mixed with other drugs, including synthetic cannabinoids or in combination with synthetic cathinones (“Wow”) (Schifano et al., 2015; Papsun et al., 2016).

Users report a slow onset of action, which possibly increases the risk of toxic overdose from redosing before peak effect is reached. Intravenous administration of MT-45 is 11 times more lethal than morphine according to data observed in mice (Helander et al., 2014; Prekupec et al., 2017). MT-45 and novel fentanyls are probably similar in addictive potential and withdrawal effects (Tracy et al., 2017).

Clinical data from 12 analytically confirmed hospital cases of MT-45 poisoning, demonstrate that, similarly to other opioids, the main dangerous effects of MT-45, are respiratory depression, cognitive deficits, and loss of consciousness. A few users reported bilateral hearing loss and significant auditory symptoms with transient tinnitus, whilst a pronounced sensorineural hearing loss still present at 2 weeks follow-up affected one user. Hence MT-45 may be an ototoxic substance (Helander et al., 2014; Lindeman et al., 2014; Papsun et al., 2016; Dolengevich-Segal et al., 2017).

Other side effects, unclearly attributable solely on MT-45 or another contaminant, include folliculitis and dermatitis with hair loss, dry eyes, elevated liver enzymes, leukonychia striata (Mees’ lines), typically found in thallium poisoning, and severe bilateral cataracts requiring surgery (Armenian et al., 2017b; Helander et al., 2017b). In a case report, autopsy revealed brain and hemorrhagic pulmonary edema and hyperemia of the internal organs (Fels et al., 2017).

Administration routes of MT-45 are typically oral or by nasal aspiration, but also intravenous, sublingual, intrarectal, or inhaled (vaporized). Typical doses reported by users are 15–30 mg for insufflation and 25–75 mg for oral administration; desired effects can last for up to 2 h (Zawilska and Andrzejczak, 2015). The effects sought by users is a sensation of well-being, relaxation and euphoria. In Switzerland 30 fatalities and several acute intoxications have been recently reported (Dolengevich-Segal et al., 2017).

Another piperazine, AD-1211 (1-(3-methyl-2-butenyl)-4-[(1R)-1-phenyl-2-(3-hydroxyphenyl)ethyl]piperazine), was also synthesized in the 1970s by the same Japanese laboratory which created MT-45. This compound has narcotic and analgesic antagonist activities with a physical dependence weaker than that of pentazocine (an opioid painkiller) (Natsuka et al., 1987). Information on this compound is limited and no pharmacotoxicological properties have been reported in the international literature.

## Conclusion

Novel synthetic opioids were originally synthesized by pharmaceutical companies in their research for analgesic drugs without addictive properties. However, because of their toxicity or abuse potential, the NSOs reported in this review were never approved for medical use. Currently NSOs are mainly used by individuals who already used heroin, prescription opioids, or other illicit opioids looking for same opiates effects: relaxation, sedation, and euphoria. Psychonauts (from the Ancient Greek Ψυχή or psyche, which means “soul,” “spirit,” or “mind,” and ναύτης or naútēs, which means “sailor” or “navigator,” herein indicating a modern drug users seeking for altered mental states) appear also interested in experimenting the eventual peculiar effects of these NPSs.

Novel synthetic opioids are readily available on internet web sites and often used in association with other recreational drugs, leading to a public health danger in many countries worldwide. These substances have been causing severe intoxications and deaths pushing the United States and European governments to take the necessary measures to prevent their further spread.

Unfortunately, conventional drug tests do not currently detect the NSOs reported in this review. The growing number of acute intoxication cases, often associated with polyabuse, indicates that pharmacological, toxicological, and forensic research on these compounds is highly needed in order to determine their pharmacokinetic profiles, long-term effects, and effective detection methods.

## Author Contributions

All the authors searched for bibliographic material, drafted different chapter of the manuscript, and contributed substantially to manuscript intellectual content and revision.

## Conflict of Interest Statement

The authors declare that the research was conducted in the absence of any commercial or financial relationships that could be construed as a potential conflict of interest.

## Footnotes

1 http://www.bluelight.org/vb/forum.php?
